# Cyclooxygenase and Cancer: Fundamental Molecular Investigations

**DOI:** 10.3390/ijms241512342

**Published:** 2023-08-02

**Authors:** Mauro Coluccia

**Affiliations:** Department of Pharmacy—Drug Sciences, University of Bari “Aldo Moro”, Via E. Orabona 4, 70125 Bari, Italy; mauro.coluccia@uniba.it; Tel.: +39-080-544-2788

The involvement of prostaglandins in cancer was first observed in human esophageal carcinoma cells, whose invasive and metastatic potential in nude mice was found to be related to PGE_2_ and PGF_2a_ production [1]. Since then, the evaluation of prostaglandins (in particular PGE_2_) and cyclooxygenases (in particular COX-2) in distinct neoplastic diseases, along with the investigations of their effects on tumor cell biology properties and tumor progression, have resulted in a very extensive, perhaps overwhelming, scientific literature.

The topic is still of great interest (in the PubMed database, the search “cyclooxygenase and cancer” yields more than 5000 results in the last 10 years), considering its present and potential applications in medical practice. Epidemiological and preclinical evidence indeed suggests that agents with anti-inflammatory COX-targeting activity, such as aspirin and non-steroidal anti-inflammatory drugs (NSAIDs), have the potential to prevent or delay cancer initiation and improve the therapeutic efficacy of cytotoxic agents and radiotherapy, as well as targeted agents and immune checkpoint inhibitors [2,3,4].

The Special Issue “Cyclooxygenase and Cancer: Fundamental Molecular Investigations” of the International Journal of *Molecular Sciences* includes five research papers, four of which concern the effects of NSAIDs on glioblastoma [5,6,7] and prostate [8] cancer cells, and one which explores a pro-proliferative COX-2 mechanism that does not involve classical prostaglandin receptor signaling [9].

Glioblastoma multiforme (GBM) is the most common and aggressive malignant glioma, accounting for about 50% of all primary malignant brain tumors in adults [10,11]. GBM has a very poor prognosis, with a median survival rate of only 14–17 months with standard treatment including maximal safe resection, adjuvant radiotherapy, and chemotherapy with temozolomide [12]. The GBM-associated increased expression of COX-2 and PGE_2_ has long been known [13,14], and extensive data suggest that elevated COX-2 activity in tumor cells and the glioblastoma microenvironment [15] can facilitate the acquisition of cancer hallmark capabilities [16] and tumor progression. Therefore, the COX-2-PGE_2_ axis may be a potential therapeutic target for which mechanistic investigations of effects on cancer cell phenotype can provide a solid rationale.

In their research paper, Ferreira et al. [5] investigated the effects of COX-1 and COX-2 inhibition upon cell proliferation, migration, and invasive properties of human GBM cells in vitro. First, the mRNA expression of PTGS1 (COX-1) and PTGS2 (COX-2) genes was evaluated in differing grades of glioma using the GlioVis software [17] for analysis of brain tumor expression in the TCGA (The Cancer Genome Atlas) and CGGA (Chinese Glioma Genome Atlas) datasets. The expression of both COX-1 and COX-2 was found to be significantly increased in grade IV GBMs in comparison with lower-grade gliomas. Having confirmed the expression of both COX isoforms in the GBM cell lines under study, the effects of non-selective (ibuprofen) or selective (SC560 for COX-1 and NS398 for COX-2) inhibitors were investigated. Overall, the results showed that COX-2 as well as COX-1 activity is important to the normal function of GBM cells in in vitro conditions, thus suggesting a coordinated pathophysiological role of both isoforms [18] in glioblastoma. In this study, the relevance of the PGE_2_ receptors EP2 and EP4 to the control of GBM cell proliferation and migration was also identified. The concomitant pharmacological inhibition of EP2 and EP4 caused a significant decrease in cell migration, which was not reverted by exogenous PGE_2_. Finally, a poorly explored area of GBM cell biology, i.e., the control of matrix metalloproteinase (MMP) expression and activity by the prostanoid pathway, was also investigated. Interestingly, MMP2 expression was significantly positively correlated with PTGS1 (COX-1) in GBM tissue. The finding that COX-1 inhibition affects MMP2 protein expression and extracellular matrix-modifying activity of GBM cells in vitro suggests a novel therapeutic target for drug development.

In addition to the current GBM therapy, novel feasible or potential targets have recently emerged [19] and are actively investigated in clinical trials [20]. As reported in the systematic review by Da Silva et al. [21], targeted therapies in GBM clinical trials can be grouped into four categories: targeting the potential for unlimited replication, growth autonomy and migration, cell cycle and apoptosis, and angiogenesis. According to a large body of research, the anticancer activity of NSAIDs depends upon their ability to interfere with tumorigenic signaling pathways [22]. In more detail, both traditional NSAIDs and COX-2 selective inhibitors can modulate many different signaling pathways, such as NF-κB, phosphodiesterases, NSAID-activated genes (NAG-1), peroxisome proliferator-activated receptors (PPAR), the Wnt pathway, cell kinetic effects, the Akt pathway, and pro-resolving mediators [23].

The increased expression of anti-apoptotic Bcl-2 family members has been described in a wide range of solid tumors and in GBM, where the levels of anti-apoptotic BCL-xL and MCL-1 are consistently increased with respect to non-malignant cells and tissues [24].

In the research paper on the effects of the non-selective COX inhibitor indomethacin on GBM cells [6], Chang C. Y. et al. extend their previous observations on indomethacin-induced glioma apoptosis involving the ceramide/protein phosphatase 2A (PP2A)/Akt axis [25]. The authors show that indomethacin can induce oxidative stress and endoplasmic reticulum (ER) stress, as well as Ask1 and p38 activation, in glioma cells. Interestingly, mechanistic investigations further indicated that the oxidative stress/ER stress/Ask1/p38 cascade is an alternative regulator of the PP2A/Akt axis, resulting in Mcl-1 and FLIP downregulation and eventually glioma apoptosis.

Even aspirin can induce tumor cell apoptosis, in most cases involving the anti-apoptotic Mcl-1 protein downregulation [26,27,28,29]. In their second paper in this Special Issue, Chang C. Y. et al. investigate the apoptotic potential of aspirin towards GBM, focusing on the molecular bases of crosstalk between anti-apoptotic and pro-apoptotic Bcl-2 family proteins and underlying apoptotic programs [7]. The authors show that the glioma cell-killing effects of aspirin involve mitochondria-mediated apoptosis accompanied by ER stress, Noxa upregulation, Mcl-1 downregulation, Bax mitochondrial distribution and oligomerization, and caspase activation. Data from genetic and pharmacological studies reveal that the axis of ER stress comprises an apoptotic cascade leading to Noxa upregulation and apoptosis. Importantly, the apoptotic programs and mediators triggered by aspirin in glioma cells were duplicated in tumor-bearing BALB/c nude mice. These findings, along with the previously reported involvement of ER stress in indomethacin-induced Mcl-1 downregulation, support ER stress as a valuable target for intervention in glioma cell apoptosis [30].

Prostate cancer (PCa) is the most common tumor in men, and it has an increasing incidence worldwide due to an aging population and increased detection [31]. Since androgens regulate prostate cancer growth, androgen deprivation therapy (ADT) is the first-line approach for advanced PCa. However, the duration of the ADT response is limited (18–24 months), and most patients progress to the more aggressive castration-resistant prostate cancer (CRPC). Multiple mechanisms for castration resistance have been proposed [32], including immune and inflammatory signaling in both cancer cells and the tumor microenvironment [33].

In their research paper [8], Benelli et al. investigated the effects of the COX-2 inhibitor celecoxib on two androgen-resistant LNCaP sublines (i.e., PDB and MDB), which recapitulate some phenotypic features of PCa evolution to CRPC [34]. PDB cells mimic the clinical condition in which cancer cells are partially exposed to androgens, whereas MDB cells mimic the clinical condition in which cancer cells survive despite a completely hormone-deprived microenvironment. Benelli et al. demonstrate that constitutive activation of ErbB family receptors controlling AKT/AR/GSK3β/P38/NF-κB and hnRNP K signaling nodes emerges in PCa cells during the progression to CRPC. Importantly, bioinformatic analyses of human prostate cancer datasets support the relevance of these pathways in PCa progression. All these molecules are simultaneously modulated by celecoxib treatment. Celecoxib reduced cell growth and induced apoptosis through AKT blockade, cleavage of poly (ADP-ribose) polymerase-1 (PARP-1), and proteasomal degradation of the anti-apoptotic protein Mcl-1. Epidermal growth factor receptor (EGFR), ErbB2, and ErbB3 degradation, and heterogeneous nuclear ribonucleoprotein K (hnRNP K) downregulation further amplified the inhibition of androgen signaling. Moreover, celecoxib reduced the invasive phenotype of CRPC cells by modulating NF-κB activity and reduced tumor growth in mouse xenografts when administered in association with the anti-EGFR receptor antibody cetuximab, thus suggesting a novel therapeutic strategy to hinder signal transduction during CRPC progression.

In the last paper of this Special Issue, Saadi et al. explore an unexpected and surprising biological property of the COX-2 protein, independent from its enzymatic activity. Based on previous data that shows that the continued exposure of COX-2 to arachidonic acid leads to the appearance of lower molecular weight COX-2 fragments [35], in their research paper [9], Saadi et al. confirm the presence of COX-2 immunoreactive fragments in a murine model of glioblastoma as well as in patient-derived colorectal cancer tissues. To provide proof of principle that COX-2 fragments can have biological effects independently from enzymatic activity, a COX-2 mutant that undergoes spontaneous cleavage was used. The K598R point mutation (i.e., arginine for lysine) at the carboxyl-terminus of COX-2 causes the occurrence of several COX-2 immunoreactive fragments in nuclear compartments and significantly enhances cell proliferation. From a mechanistic point of view, transcriptomic analyses show that K598R COX-2 significantly affects the expression of genes involved in RNA metabolism, and subsequent proteomics suggest that it is associated with proteins that regulate mRNA processing. The authors report a similar increase in proliferation by expressing just that catalytic domain of COX-2 (ΔNT-COX-2), which is completely devoid of catalytic activity in the absence of its other domains. Moreover, they show that the ΔNT-COX-2 protein also interacts in the nucleus with β-catenin, a central regulator of gene transcription. Together, these data strongly suggest that the cleavage products of COX-2 can affect cell proliferation through mechanisms that are independent of prostaglandin synthesis. Overall, the results provide a possible explanation for the poor therapeutic efficacy of NSAIDs in tumors where COX-2 expression clearly correlates with a worse prognosis. Additional studies on COX-2 fragments in human tumors, as well as on the mechanisms underlying their production and effects on tumor cell biology, will help to better understand this unexpected biological property of COX-2.
